# Right and left atrial metastasis of renal cell carcinoma: A case report

**DOI:** 10.1016/j.ijscr.2022.107692

**Published:** 2022-09-20

**Authors:** Emmanuel Luciano, Mohamed K. Kamel, Bakri Kaakeh

**Affiliations:** a Department of Surgery, Central Michigan University College of Medicine, United States of America; b Department of Cardiothoracic Surgery, Covenant Healthcare, United States of America

**Keywords:** Case report, Atrial metastasis, Cardiac metastasis, Renal cell carcinoma

## Abstract

**Introduction and importance:**

Cardiac tumors are uncommon with an estimated incidence of 0.002–0.3 % in autopsy series. Most cardiac tumors are metastatic in nature. Renal cell carcinoma (RCC) metastatic to the heart without inferior vena cava (IVC) contiguous involvement is extremely rare with about 31 cases reported in the literature and only one case with bilateral atrial metastases.

**Case presentation:**

In this report, the surgical management of metachronous RCC involving the right and left atrium is described in a 41-year-old male patient three years after initial diagnosis who presented with worsening episodes of cough, dyspnea, chest pain and hemoptysis. Transesophageal echocardiogram revealed significant inflow obstruction. The patient underwent bilateral atrial mass excision via median sternotomy. The postoperative period was unremarkable, and the patient was referred to medical oncology to pursue further treatment.

**Clinical discussion:**

Among the reported cases of cardiac RCC metastases without contiguous IVC involvement, bilateral atrial metastases are exceedingly rare. To our knowledge, this is the first case with bilateral atrial involvement to undergo surgical resection reported in the literature.

**Conclusion:**

Isolated biatrial cardiac metastases from RCC can be successfully resected with good outcomes in selected patients.

## Highlights

• Isolated biatrial metastases of renal cell carcinoma are rare.
• Successful surgical resection is possible in selected cases.
• Further oncology treatment is required.

## 1 Introduction and importance

Cardiac tumors are infrequently encountered with an estimated incidence of 0.002–0.3 % in autopsy series [1]. Over 95 % of cardiac tumors are secondary (metastatic) tumors with melanoma, malignant germ cell tumors and leukemia being the most common primary sites [1], [2], [3], [4]. Direct extension of renal cell carcinoma (RCC) to the heart via the inferior vena cava (IVC) has been documented in about 1 % of patients with RCC [5], [6], [7]. The case described in this report of RCC spreading to the cardiac chambers without IVC contiguous involvement is exceedingly rare with about 31 cases previously reported in the literature [7], [8]. Of these reported cases, isolated right ventricular metastases were the most common, with only one case of biatrial involvement which was managed non-operatively due to the poor performance status and frailty of the patient [8], [9].

In this report, the diagnosis and operative management of metastatic RCC to the right and left cardiac atria, three years after the diagnosis of the primary tumor is described. This work has been reported in line with the SCARE 2020 criteria [10].

## 2 Case presentation

**Image 1:**
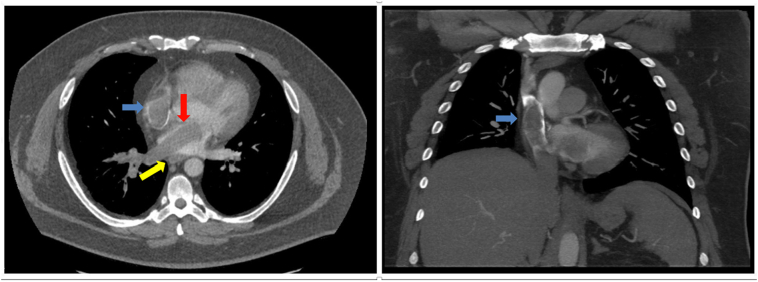
Axial and coronal view CTA revealing filling defect in the left atrium (yellow arrow), right pulmonary vein and artery (red arrow), and SVC (blue arrows).

A 41-year-old male patient presented with worsening episodes of cough, dyspnea, chest pain and hemoptysis for the past 3 months. Three years prior he had undergone a right nephrectomy with IVC thrombectomy for a T3N0M0 renal cell carcinoma of clear cell type. A computed tomography angiography (CTA) of the chest was obtained revealing a filling defect in the left atrium, right pulmonary vein and artery, and superior vena cava (SVC) (Image 1).

**Image 2:**
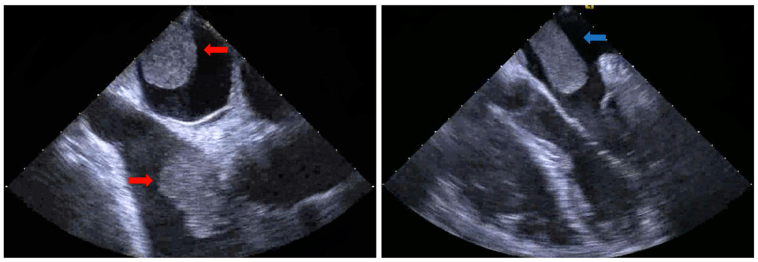
Transesophageal echocardiography. Left: bi-atrial view with both atrial masses (red arrows). Right: left atrial mass (blue arrow).

*Trans*-esophageal echocardiogram (TEE) was then performed and showed two intracardiac masses. The first was involving the right pulmonary vein and was extending into the left atrium, measuring 4.30 × 1.96 cm. The second mass involved the right atrium and SVC, measured 6.02 × 3.27 cm, and appeared to cause inflow obstruction of the SVC (Image 2). Given the patient's history of RCC and the number and location of the intracardiac masses, metastatic lesions of RCC primary were presumed. Computed tomography (CT) of the head, abdomen and pelvis was negative for any other metastatic lesions.

**Image 3:**
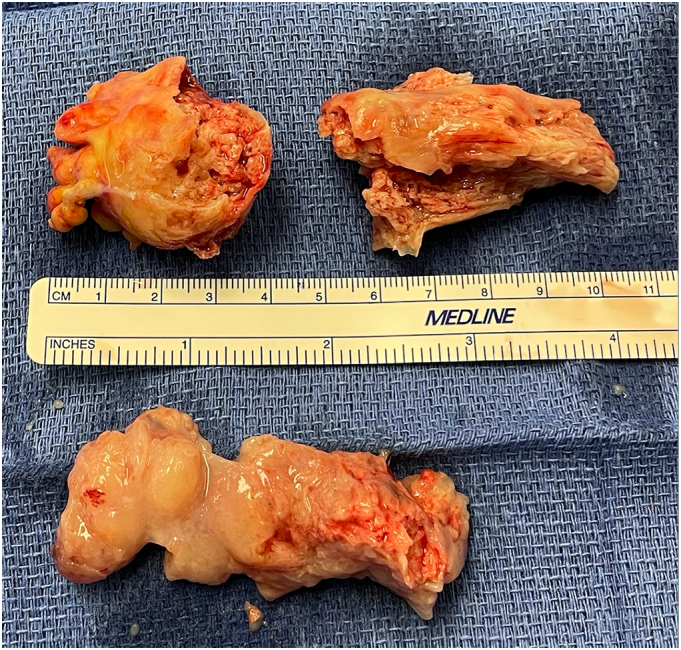
Right atrial mass resected in 2 pieces (above the ruler) and the left atrial mass resected in one piece (below the ruler).

Due to significant inflow obstruction by the tumors, surgical resection was deemed necessary to relieve the patient's symptoms. The patient did not have any comorbidities that would preclude surgery and his EuroSCORE II was calculated at 1.68 %. The procedure was performed by the senior cardiac surgery attending in an academic hospital. A standard median sternotomy was made. Aortic, SVC, IVC and retrograde cardioplegia cannulas were placed. The patient was put on cardiopulmonary bypass (CPB) with deep hypothermic circulatory arrest (DHCA). The right atrium was opened, and the right atrial mass was identified and resected. The area of the pedicle was debrided. Next, the interatrial septum was opened through the fossa ovalis, and the left atrial mass was resected. This pedicle was debrided from the wall of the right pulmonary vein. Both atrial cavities were irrigated with cold 0.9 % normal saline solution to ensure that no floating pieces of the tumor were left behind. The interatrial septum was closed using continuous 3–0 prolene suture and the right atrium was closed with continuous 4–0 prolene. The right atrial mass measured 5.5 × 5.0 × 1.5 cm and the left atrial mass 7.5 × 5.5 × 1.3 cm (image 3). The patient was then weaned off of CPB. Total CPB time was 105 min, and the aortic cross clamp time was 65 min. The patient had an uneventful postoperative recovery and was discharged home on postoperative day 5. Final pathology for both specimens was consistent with clear cell type RCC with rhabdoid features. Postoperative follow-up revealed no complications, and the patient was referred to medical oncology to pursue further treatment with immunotherapy.

## 3 Clinical discussion

Metastatic cardiac tumors are 20 to 40 times more common than primary cardiac tumors. Most secondary tumors of the heart originate from primary melanoma, lung cancer, breast cancer or lymphoma. Metastases to the heart may occur via three proposed mechanisms: hematogenous spread, direct invasion, or extension to the right atrium secondary to IVC involvement [5], [6].

Among the reported cases of cardiac RCC metastases without contiguous IVC involvement, isolated right ventricular involvement appears to be more frequent than right or left atrial involvement. While bilateral atrial metastasis is exceedingly rare. To our knowledge, this is only the second reported case of bilateral atrial metastasis from RCC, and the first case to undergo surgical resection reported in the literature [8], [9], [11]. Biatrial metastasis has been described for other metastatic tumors such as uterine melanoma and cutaneous squamous cell carcinoma; however, these cases did not undergo resection due to poor performance statuses, making this case report the first to describe the surgical resection of biatrial metastatic cardiac tumors across all primary malignancies [12], [13].

The majority of cardiac metastases from RCC reported so far have been treated with surgical excision. CPB with DHCA through median sternotomy is the most common surgical technique. Other treatment options for unresectable metastases or for frail patients include systemic treatment with targeted therapy. Limited reports have been published with the use of tyrosine kinase inhibitors (sunitinib and pazopanib) or mammalian target of rapamycin (mTOR) inhibitors. These described some regression of cardiac metastases from RCC with improvement of symptoms [7], [8]. Of note, following resection, our patient was referred to medical oncology and was started on immunotherapy with Ipilimumab and Nivolumab.

## 4 Conclusion

Isolated biatrial cardiac metastases from RCC can be successfully resected with good outcomes in selected patients. Immunotherapy is an option for unresectable metastases or for frail patients.

Consent.

Written informed consent was obtained from the patient for publication of this case report and accompanying images. A copy of the written consent is available for review by the Editor-in-Chief of this journal on request.

## Sources of funding

No external funding was available for this study.

## Ethical approval

None required.

## Research registration

N/a.

## Guarantor

Emmanuel Luciano, MD

## Provenance and peer review

Not commissioned, externally peer-reviewed.

## CRediT authorship contribution statement

Emmanuel Luciano, MD; conceptualization, methodology, writing original draft and final review and editing.

Mohamed K Kamel, MD; writing original draft, review and editing.

Bakri Kaakeh, MD; final review and editing.

## Declaration of competing interest

None declared.

## References

[1] Ren D.Y., Fuller N.D., Gilbert S.A.B., Zhang Y. (2017). Cardiac tumors: clinical perspective and therapeutic considerations. Curr. Drug Targets.

[2] Rahouma M., Arisha M.J., Elmously A. (2020). Cardiac tumors prevalence and mortality: a systematic review and meta-analysis. Int. J. Surg..

[3] Lamba G., Frishman W.H. (2012). Cardiac and pericardial tumors. Cardiol. Rev..

[4] Taguchi S. (2018). Comprehensive review of the epidemiology and treatments for malignant adult cardiac tumors. Gen. Thorac. Cardiovasc. Surg..

[5] Shah S., Vinod P., Khayata M., Lane J.L., Hegde V., Raina R. (2018). Atrial metastasis of renal cell carcinoma: a rare presentation. Cardiol. Res..

[6] Sahin S., Karatas F., Hacioglu M.B., Aytekin A., Cilbir E., Conkbayir I. (2018). Renal cell carcinoma presenting with heart metastasis without inferior vena caval and right atrial involvement. J. Cancer Res. Ther..

[7] Nkengurutse G., Wang Q., Tian F., Jiang S., Zhang L., Sun W. (2019). Renal cell carcinoma metastasizing to left atrium with coronary sinus invasion: a rare site of metastasis mimicking myxoma. Front Oncol..

[8] Ohba K., Miyata Y., Mitsunari K., Matsuo T., Mochizuki Y., Sakai H. (2014). Left atrial metastasis of renal cell carcinoma: a case report and review of the literature. BMC Res Notes.

[9] Rohani M., Roumina S., Saha S.K. (2005). Renal adenocarcinoma with intramyopericardial and right atrial metastasis, latter via coronary sinus: report of a case. Echocardiography.

[10] Agha R.A., Franchi T., Sohrabi C., Mathew G., Kerwan A., Group S.C.A.R.E. (2020). The SCARE 2020 guideline: updating consensus Surgical CAse REport (SCARE) guidelines. Int J Surg..

[11] Butany J., Leong S.W., Carmichael K., Komeda M. (2005). A 30-year analysis of cardiac neoplasms at autopsy. Can. J. Cardiol..

[12] Geredeli C., Boruban M.C., Poyraz N., Artac M., Aribas A., Koral L. (2015). Biatrial cardiac metastases in a patient with uterine cervix malignant melanoma. Case Rep. Cardiol..

[13] Dottorini L., Sarno I., Scopelliti P. (2019). Case report of a 65-year-old man with biatrial metastatic localisation from poorly differentiated cutaneous squamous cell carcinoma. Ecancermedicalscience.

